# An explainable web application based on machine learning for predicting fragility fracture in people living with HIV: data from Beijing Ditan Hospital, China

**DOI:** 10.3389/fcimb.2025.1461740

**Published:** 2025-03-14

**Authors:** Bo Liu, Qiang Zhang, Xin Li

**Affiliations:** ^1^ Department of Orthopaedics, Beijing Ditan Hospital, Capital Medical University, Beijing, China; ^2^ National Center for Infectious Diseases, Beijing, China

**Keywords:** fragility fracture, PLWH, web calculator, machine learning, XGBoost, SHAP, risk assessment

## Abstract

**Purpose:**

This study aimed to develop and validate a novel web-based calculator using machine learning algorithms to predict fragility fracture risk in People living with HIV (PLWH), who face increased morbidity and mortality from such fractures.

**Method:**

We retrospectively analyzed clinical data from Beijing Ditan Hospital orthopedic department between 2015 and September 2023. The dataset included 1045 patients (2015-2021) for training and 450 patients (2021-September 2023) for external testing. Feature selection was performed using multivariable logistic regression, LASSO, Boruta, and RFE-RF. Six machine learning models (logistic regression, decision trees, SVM, KNN, random forest, and XGBoost) were trained with 10-fold cross-validation and hyperparameter tuning. Model performance was assessed with ROC curves, Decision Curve Analysis, and other metrics. The optimal model was integrated into an online risk assessment calculator.

**Results:**

The XGBoost model showed the highest predictive performance, with key features including age, smoking, fall history, TDF use, HIV viral load, vitamin D, hemoglobin, albumin, CD4 count, and lumbar spine BMD. It achieved an ROC-AUC of 0.984 (95% CI: 0.977-0.99) in the training set and 0.979 (95% CI: 0.965-0.992) in the external test set. Decision Curve Analysis indicated clinical utility across various threshold probabilities, with calibration curves showing high concordance between predicted and observed risks. SHAP values explained individual risk profiles. The XGBoostpowered web calculator (https://sydtliubo.shinyapps.io/cls2shiny/) enables clinicians and patients to assess fragility fracture risk in PLWH.

**Conclusion:**

We developed a web-based risk assessment tool using the XGBoost algorithm for predicting fragility fractures in HIV-positive patients. This tool, with its high accuracy and interpretability, aids in fracture risk stratification and management, potentially reducing the burden of fragility fractures in the HIV population.

## Introduction

Fragility fractures, characterized by low-energy trauma and decreased bone strength, pose a significant burden on individuals living with HIV (PLWH) (Womack et al., 2011; Biver, 2022). Despite advancements in antiretroviral therapy (ART) and improved life expectancy, PLWH experience a higher prevalence of fragility fractures compared to the general population (Shiau et al., 2013; Hoy and Young, 2016). These fractures, particularly those involving the hip, vertebrae, and wrist, are associated with increased morbidity, mortality, and substantial healthcare costs (Althoff et al., 2016). The increased risk of fragility fractures in PLWH is multifactorial, involving a complex interplay of traditional risk factors, HIV-related factors, and antiretroviral therapy (ART) effects (Yong et al., 2011). Traditional risk factors, such as advanced age, low body mass index, smoking, and alcohol consumption, play a role. Additionally, HIV-related factors, including chronic inflammation, immune dysregulation, vitamin D deficiency, and potential direct effects of the virus on bone metabolism, contribute to the increased fracture risk (Ahmed et al., 2023). Certain antiretroviral drugs, particularly tenofovir disoproxil fumarate (TDF), have been associated with bone mineral density (BMD) loss and increased fracture risk (Ahmed et al., 2023). Identifying and stratifying PLWH at high risk for fragility fractures is crucial for implementing targeted prevention and management strategies. Early intervention, such as lifestyle modifications, calcium and vitamin D supplementation, and pharmacological therapies, can potentially reduce the burden of fragility fractures in this vulnerable population.

Several fracture risk assessment tools have been developed and widely used in clinical practice, primarily for the general population. The Fracture Risk Assessment Tool (FRAX), developed by the World Health Organization (WHO), is one of the most commonly used tools (McGee and Cotter, 2024). FRAX calculates the 10-year probability of hip fracture and major osteoporotic fracture based on clinical risk factors, with or without BMD measurements (Stephens et al., 2016). Other fracture risk assessment tools include the QFracture score, which incorporates additional risk factors such as falls, diabetes, and medications (Kanis et al., 2016), and the Garvan Fracture Risk Calculator, which accounts for the number of falls and BMD measurements at multiple sites (van den Bergh et al., 2010). While these existing tools have proven valuable in fracture risk assessment, they have limitations when applied to the HIV population. FRAX and other tools were developed and validated primarily in the general population, failing to account for the unique risk factors and characteristics of PLWH. Factors such as HIV-related inflammation, immune dysregulation, and ART effects are not explicitly considered in these tools, potentially leading to inaccurate risk estimates for PLWH. Furthermore, the majority of existing tools rely heavily on BMD measurements, which may underestimate fracture risk in PLWH (Yang et al., 2018). PLWH can experience fractures at higher BMD levels compared to the general population, suggesting that factors beyond BMD play a significant role in fracture risk assessment for this population. Given these limitations, there is a critical need for tailored fracture risk assessment tools that incorporate HIV-specific risk factors and leverage advanced analytical techniques to accurately predict fracture risk in PLWH. Machine learning algorithms offer a promising approach to address this need, as they can handle complex, non-linear relationships and incorporate a wide range of relevant risk factors (Vizcarra et al., 2023).

Machine learning algorithms have gained significant attention in various fields, including healthcare, due to their ability to uncover complex patterns and relationships within large datasets. In the context of fracture risk prediction, machine learning approaches offer several advantages over traditional statistical methods. Firstly, machine learning algorithms can effectively handle non-linear relationships and high-dimensional data, which are often present in fracture risk assessment scenarios (Kong et al., 2020). Traditional logistic regression models may oversimplify these complex relationships, leading to suboptimal performance. Secondly, machine learning algorithms can incorporate a wide range of risk factors, including demographic, clinical, biochemical, and imaging data, without making strong assumptions about their distributions or interactions. This flexibility allows for a more comprehensive assessment of fracture risk, capturing the intricate interplay of various risk factors (Shim et al., 2020). Thirdly, certain machine learning algorithms, such as ensemble methods (e.g., random forests, gradient boosting), have demonstrated superior predictive performance in fracture risk prediction tasks compared to traditional models. These algorithms can effectively capture complex patterns and handle non-linear relationships, potentially improving the accuracy of fracture risk estimates. While machine learning has shown promising applications in fracture risk prediction for the general population, its potential in the context of PLWH remains largely unexplored (Vizcarra et al., 2023). The unique risk factors and characteristics of PLWH necessitate the development of tailored machine learning models that can accurately capture the nuances of fracture risk in this population.

The primary objective of this study is to develop and validate a novel web-based calculator powered by machine learning algorithms to predict the risk of fragility fractures in PLWH. By leveraging the strengths of machine learning techniques and incorporating HIV-specific risk factors, this tool aims to provide accurate and personalized fracture risk assessments for individuals living with HIV. Secondarily, this study seeks to identify the key risk factors associated with fragility fractures in PLWH, evaluate the performance of various machine learning models, and provide interpretable predictions to aid clinical decision-making. Understanding the relative importance of different risk factors and their interactions can inform targeted prevention and management strategies for this vulnerable population. The significance of this study lies in its potential to improve fracture risk stratification and management for PLWH. By accurately identifying individuals at high risk for fragility fractures, clinicians can implement timely interventions, such as lifestyle modifications, targeted pharmacological therapies, and close monitoring. This proactive approach may ultimately reduce the burden of fragility fractures in PLWH, mitigating associated morbidity, mortality, and healthcare costs. Furthermore, the development of a user-friendly, web-based calculator can facilitate the integration of advanced fracture risk assessment into clinical practice, empowering healthcare professionals and patients to make informed decisions regarding fracture prevention and management.

## Materials and methods

### Study design and participants selection

**Figure 1:**
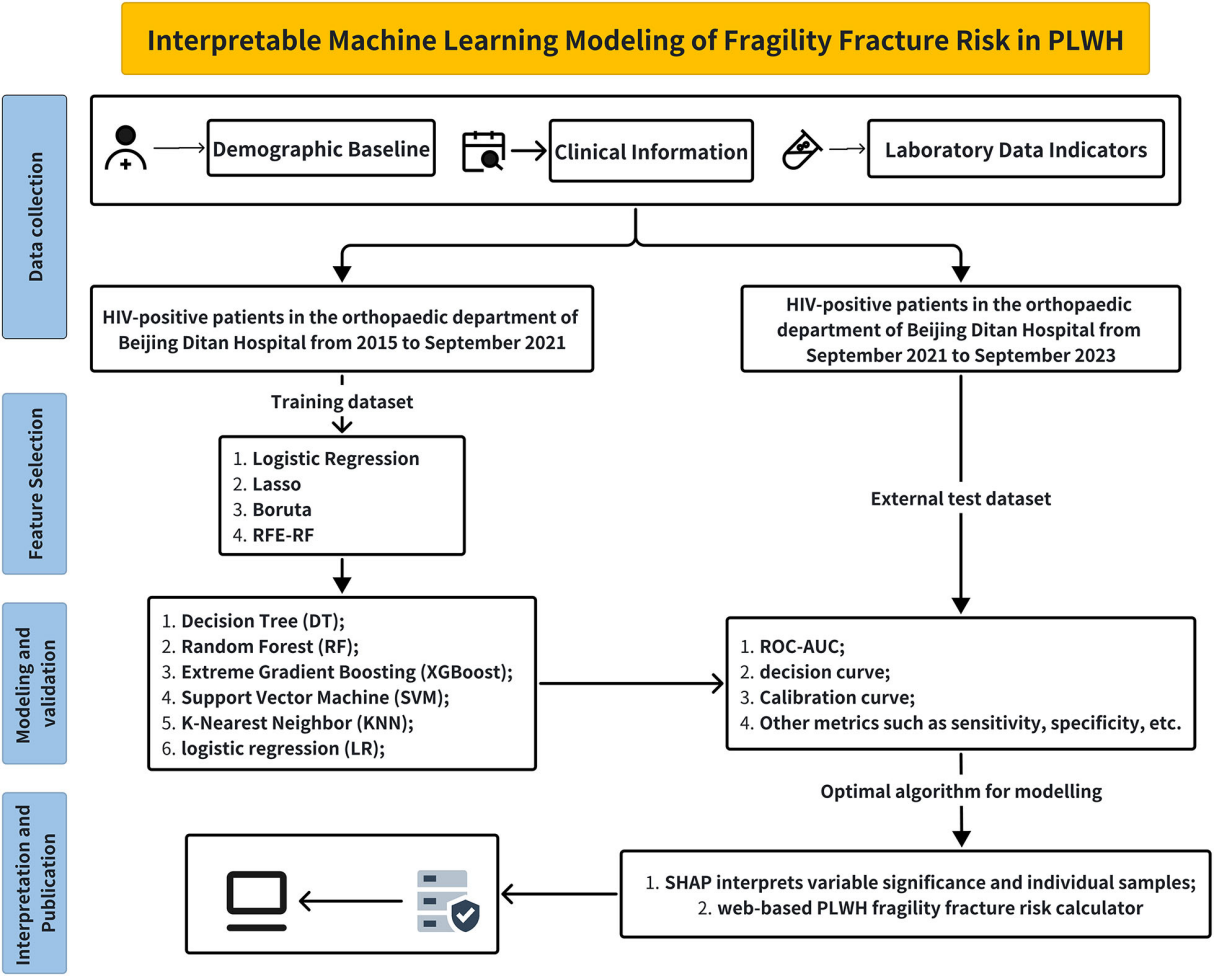
Flowchart of this study.

A retrospective study was conducted using data obtained from the orthopedic department of Beijing Ditan Hospital. The study period spanned from 2015 to September 2023. Clinical data variables were collected, including demographic information, medical history, medication use, laboratory results, and bone mineral density measurements. The dataset was split into a training set (1045 patients, 2015-2021) and an external test set (450 patients, 2021-September 2023). **Figure 1** outlines the overall methodology of this study.

Inclusion and exclusion criteria were as follows: Inclusion Criteria: 1) Adults aged 18 years or older with a confirmed diagnosis of HIV infection, as per the guidelines set by the Chinese Center for Disease Control and Prevention (China CDC); 2) Patients admitted to the orthopedic departments of Beijing Ditan Hospital from 2015 to 2023; 3) Patients diagnosed with fragility fractures, regardless of the anatomical site; 4) Patients with available bone mineral density measurements and clinical data related to bone health and fracture risk; 5) Patients who provided written informed consent to participate in the study.

Exclusion Criteria: 1) Patients with incomplete clinical data, including missing information on co-morbidity or essential bone mineral density parameters; 2) Patients with multiple fractures or pathological fractures not related to fragility; 3) Patients with severe comorbidities or opportunistic infections, such as Pneumocystis pneumonia, tuberculosis, toxoplasmosis, Candida albicans, Kaposi’s sarcoma, or other conditions that could significantly impact bone health or study outcomes; 4) Patients who declined to participate or withdrew consent during the study period.

### Data collection

The extensive set of variables considered in this study was carefully curated based on existing literature and clinical expertise, aiming to capture the multifactorial nature of fracture risk in PLWH. These include demographic factors like age, gender, and menopause status, as well as lifestyle factors like smoking and alcohol consumption, which can decrease bone mineral density (SChinas et al., 2024). HIV infection itself, its duration, and certain antiretroviral therapies can adversely affect bone metabolism through mechanisms such as inflammation and drug-induced deficiencies (Biver, 2022). Comorbidities like hypertension, diabetes, and hepatitis B/C contribute to bone loss and fracture risk through impaired bone turnover and chronic inflammation (Biver et al., 2017). Fall history and corticosteroid use are also significant risk factors (Yin and Falutz, 2016; Womack et al., 2023). Laboratory parameters like bone metabolism markers, complete blood count, liver/kidney function tests, and bone mineral density measurements were included to assess overall health, bone turnover, and fracture risk in PLWH (Yin and Falutz, 2016). To ensure data accuracy, two independent physicians reviewed and extracted clinical data from records, minimizing biases.

### Blood sample collection and processing

For the collection of fasting blood samples, Vacutainer tubes containing EDTA (Becton Dickinson, Franklin Lakes, NJ, USA) were used for venipuncture. These tubes were specifically chosen for flow cytometry analysis and morphological examination. To ensure proper clotting, serum samples were allowed to stand for 45 minutes before undergoing centrifugation at 3000 g for 10 minutes. After centrifugation, serum aliquots were carefully maintained at a cooled temperature of -80°C.

### HIV diagnosis, HIV viral load measurement and T lymphocyte count

The diagnosis of HIV was established using the gold standard HIV-1/2 antibody testing, which involved the utilization of enzyme-linked immunoassay (ELISA) and rapid methods conducted by our hospital’s laboratory doctors. Various equipment and reagents were employed, including the 4th generation HIV kit (Abbott, UK), detection reagent: Murex HIV Ag/Ab, mini-VIDSA analyzer, Bio-Rad MODEL1575 plate washer, Axsym chemiluminescent immunoassay analyzer (Abbott, UK), and ELECYS2010 chameleon enzyme immunoassay apparatus (Roche, Switzerland).

To quantify plasma viral load, the Abbott RealTime HIV viral load assay (m2000sp) from Abbott Molecular, IL, USA, was utilized. This assay has a sensitivity threshold of 40 copies/mL. For the determination of absolute CD4 cell counts in whole blood, standard flow cytometry was performed using the Beckman Coulter Navios device (Beckman, San Jose, CA, USA).

Fluorochrome-tagged monoclonal antibodies supplied by BD Biosciences, San Jose, CA, were used for the characterization of freshly isolated cell phenotypes and the identification of T cell phenotypes. Specifically, anti-CD4 FITC (clone RPA-T4, RRID: AB_2562052) and anti-CD8AF700 (clone RPA-T8, RRID: AB_396953) antibodies were employed. The cells were incubated with these antibodies for 15 minutes at room temperature in the dark, followed by washing and analysis on a Beckman Coulter Navios flow cytometer (Beckman, San Jose, CA, USA). T helper and cytotoxic T cells were identified by their positive surface expression of CD4 and CD8, respectively, with their percentages reported relative to the gated total lymphocyte population.

### Lumbar spine, left femoral neck, hip bone mineral density measurement

Bone Mineral Density (BMD) was measured using dual-energy X-ray absorptiometry (DXA) with the HOLOGIC Discovery Wi (Hologic Inc., Marlborough, MA, USA). The lumbar spine (L1-L4) and proximal femur (total hip and femoral neck) were scanned. BMD results were expressed as grams per square centimeter (g/cm²) and T-scores or Z-scores as appropriate. For patients under 50 years of age, Z-scores were used to assess BMD: Normal: Z-score > -2.0; Low bone mass: Z-score ≤-2.0; For patients 50 years and older, the World Health Organization (WHO) criteria were applied using T-scores: Normal: T-score≥-1.0; Osteopenia: T-score between -1.0 and -2.5; Osteoporosis: T-score≤-2.5. Regular calibration and quality control procedures were followed to ensure accuracy. BMD data were integrated into the study database for analysis. Accurate BMD assessment is critical in the PLWH, who are at increased risk for bone density loss and fragility fractures, especially those on TDF.

### Definition of fragility fractures in PLWH

Fragility fractures, which are the outcome of interest in studies involving in PLWH, are defined as fractures that occur as a result of minimal trauma or low-energy injuries, such as a fall from standing height or less (Womack et al., 2021; Vizcarra et al., 2023). These fractures are typically associated with decreased bone mineral density (BMD) and reduced bone strength, making individuals more susceptible to fractures even with minimal force. Fragility fractures can occur in various bones, including the vertebrae (spine), hip, wrist, and others. They are often indicative of underlying conditions such as osteoporosis or low bone mass, which may be exacerbated in PLWH due to factors like chronic inflammation, ART use, hormonal imbalances, and lifestyle factors contributing to accelerated bone loss and increased fracture risk. These fractures can have significant consequences, including pain, disability, loss of independence, and increased mortality. Therefore, understanding and preventing fragility fractures in PLWH is crucial for improving their overall health outcomes.

### Statistical analysis

The data were analyzed using R version 4.1.3 (https://www.R-project.org). Statistical significance was established at a P-value of less than 0.05. Continuous variables were expressed as mean ± standard deviation (SD) or interquartile range (IQR) and compared using one-way ANOVA or the Kruskal-Wallis U test based on distribution. Categorical variables were presented as percentages (n, %) and compared using the Chi-squared test or Fisher’s exact test as appropriate.

For feature selection, multivariable logistic regression, Least Absolute Shrinkage and Selection Operator (LASSO), Boruta, and Recursive Feature Elimination with Random Forest (RFE-RF) were employed. Six machine learning models were trained: logistic regression, decision trees, Support Vector Machine (SVM), K-Nearest Neighbors (KNN), random forest, and Extreme Gradient Boosting (XGBoost). The models were trained using 10-fold cross-validation and hyperparameter tuning via random grid search. Model performance was evaluated using receiver operating characteristic (ROC) curves, Decision Curve Analysis (DCA), and additional relevant metrics. The best-performing model was incorporated into a web-based risk assessment calculator designed to predict fragility fractures in PLWH. Calibration plots were utilized to assess the accuracy of the models in predicting the actual risk. Additionally, SHapley Additive exPlanations (SHAP) values were used to interpret the influence of each variable on the model’s predictions, providing insights into individual risk profiles. This methodological approach ensured robust model development and validation, with the final model made accessible through an online platform (https://login.shinyapps.io/) to aid clinicians and patients in evaluating fracture risk.

### Feature selection

Feature selection was a crucial step in the model-building process, aimed at identifying the most relevant subset of features for the target variable. Our study employed a comprehensive, multi-stage approach combining four different feature selection methods: multivariable logistic regression, LASSO regression, Boruta algorithm, and Random Forest-based Recursive Feature Elimination (RFE-RF).

Specifically, the process of feature screening is as follows:

1. The univariate logistic regression and multivariable logistic regression was performed, and variables with p-values less than 0.05 were selected;
2. LASSO regression were applied to further refine the feature subset. The union of variables identified by these two methods was considered for further analysis;
3. Boruta algorithm and RFE-RF, both based on Random Forest classifiers, were employed to rank the importance of the remaining features;
4. Guided by these importance scores and informed by relevant literature (Yin and Falutz, 2016; Biver, 2022; Ahmed et al., 2023; McGee and Cotter, 2024), less influential variables were eliminated, culminating in the final selection of 10 features.

By combining statistical significance, regularization techniques, ensemble methods, and domain knowledge, the most informative and predictive features were identified, enhancing the model’s performance and interpretability.

### Model development and evaluation

The machine learning algorithm models were developed using R version 4.1.3, utilizing the Tidymodels package. Tidymodels is a suite of packages designed for machine learning that adheres to tidy principles and ensures reproducibility. In this study, six machine learning algorithms—Logistic Regression (LR), Decision Tree (DT), k-Nearest Neighbors (KNN), Support Vector Machine (SVM), Random Forest (RF), and Extreme Gradient Boosting (XGBoost)—were utilized to construct the diagnostic model for fragility fractures in PLWH.

The development of the models employed the six machine learning algorithms. Each classification algorithm underwent hyperparameter tuning through 10-fold cross-validation. After selecting the optimal hyperparameters, the models were retrained on the complete training subset to finalize the final models. These final models were then assessed on the external test cohort. The evaluation of the trained models’ performance included comparing ROC curves and PR curves for both the training and external test cohorts. Additionally, Decision Curve Analysis (DCA) curves, calibration curves, and heatmaps of various metrics such as specificity, sensitivity, and other performance indicators were generated. These comprehensive evaluations ensured a thorough assessment of each model’s adequacy and efficacy.

### Interpretability and online risk assessment tools using optimal models

Model interpretation was carried out using SHAP (SHapley Additive exPlanations) values to explain the predictions of the optimal model, particularly the XGBoost model. SHAP values were used to assess the variable importance for all samples, highlighting the most influential features across the dataset. Additionally, SHAP waterfall plots were generated for individual samples, providing detailed insights into how each feature contributed to the prediction for specific cases.

Ultimately, the XGBoost model emerged as the most optimal model, displaying superior performance metrics. This model was then deployed on the ShinyApps website (https://www.shinyapps.io/), creating an accessible online computing platform. This platform enables real-time risk assessment for fragility fractures in PLWH. It is designed to be user-friendly, providing both clinicians and patients with accessible risk predictions and detailed interpretations of each prediction, thereby enhancing clinical decision-making and potentially reducing the incidence of fragility fractures in this vulnerable population.

## Results

### Characteristics and baseline of HIV-positive patients with and without fragility fractures

**Table 1** summarizes the baseline characteristics of 1,495 HIV-positive patients, categorized into non-fracture (n=1,268) and fracture groups (n=227). The fracture group was older (42.3 vs. 38.4 years, p<0.001) and had a higher prevalence of menopause (75.0% vs. 47.5%, p=0.028), diabetes (25.1% vs. 16.5%, p=0.002), and HBV/HCV co-infection (18.5% vs. 12.1%, p=0.011). This group also demonstrated higher smoking rates (p<0.001) and longer HIV infection duration (64.3 vs. 56.5 months, p=0.030). Importantly, fracture patients had lower CD4+ T-cell counts (389 vs. 572 cells/µL, p<0.001), higher HIV RNA loads (p=0.009), and poorer nutritional and bone health, indicated by lower hemoglobin (134 vs. 151 g/L, p<0.001), albumin (42.3 vs. 46.4 g/L, p<0.001), and vitamin D levels (20.4 vs. 26.2 ng/mL, p<0.001), as well as lower bone mineral densities (p<0.001 for all). These findings underscore significant differences in health status and risk factors between PLWH with and without fragility fractures, highlighting the need for targeted clinical management in the fracture group.

**Table 1:** Baseline characteristics of HIV-positive patients with and without fragility fractures.

Variable	All	No	Yes	P-value
	*N=1495*	*N=1268*	*N=227*	
Gender: Male (n, %)	1372 (91.8%)	1169 (92.2%)	203 (89.4%)	0.206
Age (Years)	38.9 (11.5)	38.4 (11.0)	42.3 (13.6)	<0.001
Menopause (n, %)	65 (52.8%)	47 (47.5%)	18 (75.0%)	0.028
BMI (kg/m^2^)	23.1 (3.46)	23.2 (3.49)	22.7 (3.26)	0.055
Smoke (n, %)				<0.001
Former	111 (7.42%)	70 (5.52%)	41 (18.1%)	
Now	187 (12.5%)	149 (11.8%)	38 (16.7%)	
Drinking (n, %)	300 (20.1%)	259 (20.4%)	41 (18.1%)	0.466
Hypertension (n, %)	174 (11.6%)	155 (12.2%)	19 (8.37%)	0.120
Diabetes (n, %)	266 (17.8%)	209 (16.5%)	57 (25.1%)	0.002
HBV_HCV (n, %)	195 (13.0%)	153 (12.1%)	42 (18.5%)	0.011
Fall_history (n, %)	387 (25.9%)	212 (16.7%)	175 (77.1%)	<0.001
Corticosteroids_used (n, %)	180 (12.0%)	150 (11.8%)	30 (13.2%)	0.631
Duration_infection (n, %)	57.7 (47.1)	56.5 (46.4)	64.3 (50.2)	0.030
TDF (n, %)	1037 (69.4%)	825 (65.1%)	212 (93.4%)	<0.001
HIV_RNA_load (n, %)				0.009
1000-100000	245 (16.4%)	192 (15.1%)	53 (23.3%)	
>100000	117 (7.83%)	101 (7.97%)	16 (7.05%)	
CD4 (cells/ul)	544 (309)	572 (315)	389 (218)	<0.001
CD8 (cells/ul)	924 (509)	951 (520)	771 (409)	<0.001
CD4 CD8_Ratio	0.68 (0.46)	0.70 (0.48)	0.58 (0.34)	<0.001
WBC (10^9^/L)	6.20 (2.05)	6.12 (1.97)	6.64 (2.40)	0.002
Hb (g/L)	148 (20.5)	151 (18.5)	134 (24.3)	<0.001
PLT (10^9^/L)	230 (65.5)	229 (62.7)	236 (79.4)	0.226
ALB (g/L)	45.8 (5.42)	46.4 (4.98)	42.3 (6.42)	<0.001
Ca (mmol/l)	2.31 (0.12)	2.31 (0.12)	2.29 (0.12)	0.037
P (mmol/l)	1.02 (0.29)	1.02 (0.30)	1.05 (0.22)	0.047
VD (ng/mL)	25.3 (6.05)	26.2 (5.93)	20.4 (3.96)	<0.001
TC (mmol/l)	4.40 (0.94)	4.39 (0.95)	4.44 (0.91)	0.496
TG (mmol/l)	1.64 (1.00)	1.63 (1.01)	1.68 (0.95)	0.523
LDL_C (mmol/l)	2.70 (1.07)	2.67 (0.82)	2.84 (1.95)	0.192
HDL_C (mmol/l)	1.12 (0.32)	1.13 (0.32)	1.08 (0.27)	0.006
UA (umol/l)	379 (100)	378 (101)	384 (97.2)	0.367
eGFR (ml/min/1.73m^2^)	108 (17.3)	109 (16.7)	103 (19.9)	<0.001
LS_BMD (g/cm3)	0.945 (0.128)	0.962 (0.124)	0.852 (0.111)	<0.001
LFN_BMD (g/cm3)	0.766 (0.123)	0.778 (0.120)	0.699 (0.117)	<0.001
Hip_BMD (g/cm3)	0.889 (0.132)	0.901 (0.130)	0.821 (0.119)	<0.001

BMI, Body Mass Index; HBV, Hepatitis B Virus; HCV, Hepatitis C Virus; TDF, Tenofovir Disoproxil Fumarate; HIV_RNA_load, HIV RNA Load; CD4, CD4 T Cells; CD8, CD8 T Cells; CD4_CD8_Ratio, CD4/CD8 Ratio; WBC, White Blood Cell Count; Hb, Hemoglobin; PLT, Platelet Count; ALB, Albumin; Ca, Calcium; P, Phosphorus; VD, Vitamin D; TC, Total Cholesterol; TG, Triglycerides; LDL_C, Low-Density Lipoprotein Cholesterol; HDL_C, High-Density Lipoprotein Cholesterol; UA, Uric Acid; eGFR, Estimated Glomerular Filtration Rate; LS_BMD, Lumbar Spine Bone Mineral Density; LFN_BMD, Left Femoral Neck Bone Mineral Density; Hip_BMD, Hip Bone Mineral Density.

### Patient characteristics for training set and external test set

**Supplementary Table 1** summarizes the baseline characteristics of 1,495 PLWH, split into training (n=1,045) and test datasets (n=450). The proportion of patients with fractures was similar in both groups (15.1% vs. 15.3%, p=0.978). The majority were male (91.8% overall), with comparable ages (38.9 years in total, p=0.192). Menopause rates (52.8% overall, p=0.313), BMI (23.1 kg/m², p=0.771), and smoking habits showed no significant differences, though smoking rates varied slightly (p=0.046). Drinking, hypertension, diabetes, HBV/HCV co-infection, fall history, and corticosteroid use were consistent across datasets. Infection duration (57.7 months, p=0.159), TDF use (69.4%, p=0.193), and HIV RNA load distributions were similar. CD4, CD8 counts, CD4/CD8 ratio, WBC, Hb, PLT, ALB, Ca, P, VD, lipid profiles, UA, eGFR, and bone mineral densities (LS BMD, LFN BMD, Hip BMD) showed no significant differences between the groups. Overall, the training and test datasets were well-matched, providing a robust basis for further analysis.

### Feature selection for model

To enhance the practicality and operability of our predictive model, we first conducted univariate logistic regression analysis on a set of potential variables to identify those significantly associated with fragility fractures in PLWH. Variables with significant p-values were then included in the multivariate logistic regression analysis to control for confounding factors. Our findings revealed that out of the initial pool of variables, the following were significant predictors in the multivariate analysis: Age, current smoking status, diabetes, history of falls, TDF usage, HIV RNA load (1000-100000 copies/mL), CD4 count, WBC, hemoglobin (Hb), albumin (ALB), vitamin D (VD), and lumbar spine bone mineral density (LS BMD). These results are summarized in **Table 2**.

**Table 2:** Univariate and multivariate logistic binary regression analyses of fragility fractures in HIV-positive patients.

Variables	Univariate binary logistic regression		Multivariate binary logistic regression	
	OR (95% CI)	P-value	OR (95% CI)	P-value
Gender (n, %)	1.40 (0.86, 2.20)	0.176		
Age (Years)	1.03 (1.02, 1.04)	<0.001	0.96 (0.93, 0.98)	**0.001**
BMI (Kg/M^2^)	0.96 (0.92, 1.00)	0.063		
Smoke (n, %)		<0.001		
Former	4.15 (2.71, 6.31)		1.45 (0.58, 3.62)	0.429
Now	1.81 (1.20, 2.66)		0.30 (0.13, 0.70)	**0.0049**
Drinking (n, %)	0.86 (0.59, 1.23)	0.408		
Hypertension (n, %)	0.66 (0.39, 1.05)	0.083		
Diabetes (n, %)	1.70 (1.21, 2.36)	0.003	0.31 (0.15, 0.66)	**0.003**
HBV_HCV (n, %)	1.65 (1.13, 2.39)	0.011	1.40 (0.67, 2.88)	0.367
Fall_history (n, %)	16.8 (12.0, 23.8)	<0.001	85.1 (42.4, 183)	**<0.001**
Corticosteroids_used (n, %)	1.14 (0.73, 1.71)	0.559		
Duration_infection (months)	1.00 (1.00, 1.01)	0.027	1.00 (0.99, 1.00)	0.294
TDF (n, %)	7.59 (4.59, 13.5)	<0.001	21.0 (9.17, 52.1)	**<0.001**
HIV_RNA_load (n, %)		0.013		
1000-100000	1.70 (1.20, 2.40)		3.05 (1.44, 6.59)	**0.004**
>100000	0.98 (0.54, 1.66)		0.45 (0.14, 1.38)	0.171
CD4 (cells/ul)	0.79 (0.74, 0.83)	<0.001	0.80 (0.66, 0.97)	**0.021**
CD8 (cells/ul)	0.91 (0.88, 0.94)	<0.001	0.97 (0.89, 1.06)	0.545
CD4/CD8 Ratio	0.49 (0.33, 0.72)	<0.001	0.62 (0.19, 1.71)	0.407
WBC (10^9^/L)	1.12 (1.05, 1.19)	<0.001	1.16 (1.05, 1.30)	**0.005**
Hb (g/L)	0.97 (0.96, 0.97)	<0.001	0.96 (0.94, 0.97)	**<0.001**
PLT (10^9^/L)	1.17 (0.94, 1.44)	0.156		
ALB (g/L)	0.88 (0.86, 0.91)	<0.001	0.95 (0.91, 0.99)	**0.013**
Ca (mmol/l)	0.29 (0.09, 0.94)	0.040	1.05 (0.98, 1.16)	0.055
P (mmol/l)	1.38 (0.90, 2.08)	0.133		
VD (ng/mL)	0.82 (0.79, 0.84)	<0.001	0.77 (0.72, 0.81)	**<0.001**
TC (mmol/l)	1.05 (0.90, 1.22)	0.510		
TG (mmol/l)	1.04 (0.91, 1.19)	0.543		
LDL_C (mmol/l)	1.12 (1.00, 1.29)	0.047	1.24 (0.89, 1.73)	0.223
HDL_C (mmol/l)	0.53 (0.32, 0.87)	0.011	0.63 (0.25, 1.50)	0.307
UA (umol/l)	1.06 (0.93, 1.22)	0.380		
eGFR (ml/min/1.73m^2^)	0.85 (0.78, 0.92)	<0.001	0.91 (0.78, 1.07)	0.281
LS_BMD (g/cm^3^)	0.47 (0.41, 0.53)	<0.001	0.48 (0.36, 0.62)	**<0.001**
LFN_BMD (g/cm^3^)	0.54 (0.47, 0.62)	<0.001	0.91 (0.61, 1.37)	0.633
Hip_BMD (g/cm^3^)	0.57 (0.50, 0.65)	<0.001	0.98 (0.66, 1.38)	0.932

OR, Odds Ratio; CI, Confidence Interval.

BMI, Body Mass Index; HBV, Hepatitis B Virus; HCV, Hepatitis C Virus; TDF, Tenofovir Disoproxil Fumarate; HIV_RNA_load, HIV RNA Load; CD4, CD4 T Cells; CD8, CD8 T Cells; CD4_CD8_Ratio, CD4/CD8 Ratio; WBC, White Blood Cell Count; Hb, Hemoglobin; PLT, Platelet Count; ALB, Albumin; Ca, Calcium; P, Phosphorus; VD, Vitamin D; TC, Total Cholesterol; TG, Triglycerides; LDL_C, Low-Density Lipoprotein Cholesterol; HDL_C, High-Density Lipoprotein Cholesterol; UA, Uric Acid; eGFR, Estimated Glomerular Filtration Rate; LS_BMD, Lumbar Spine Bone Mineral Density; LFN_BMD, Left Femoral Neck Bone Mineral Density; Hip_BMD, Hip Bone Mineral Density.

Bold implies that the p-value is statistically significant.

**Figure 2:**
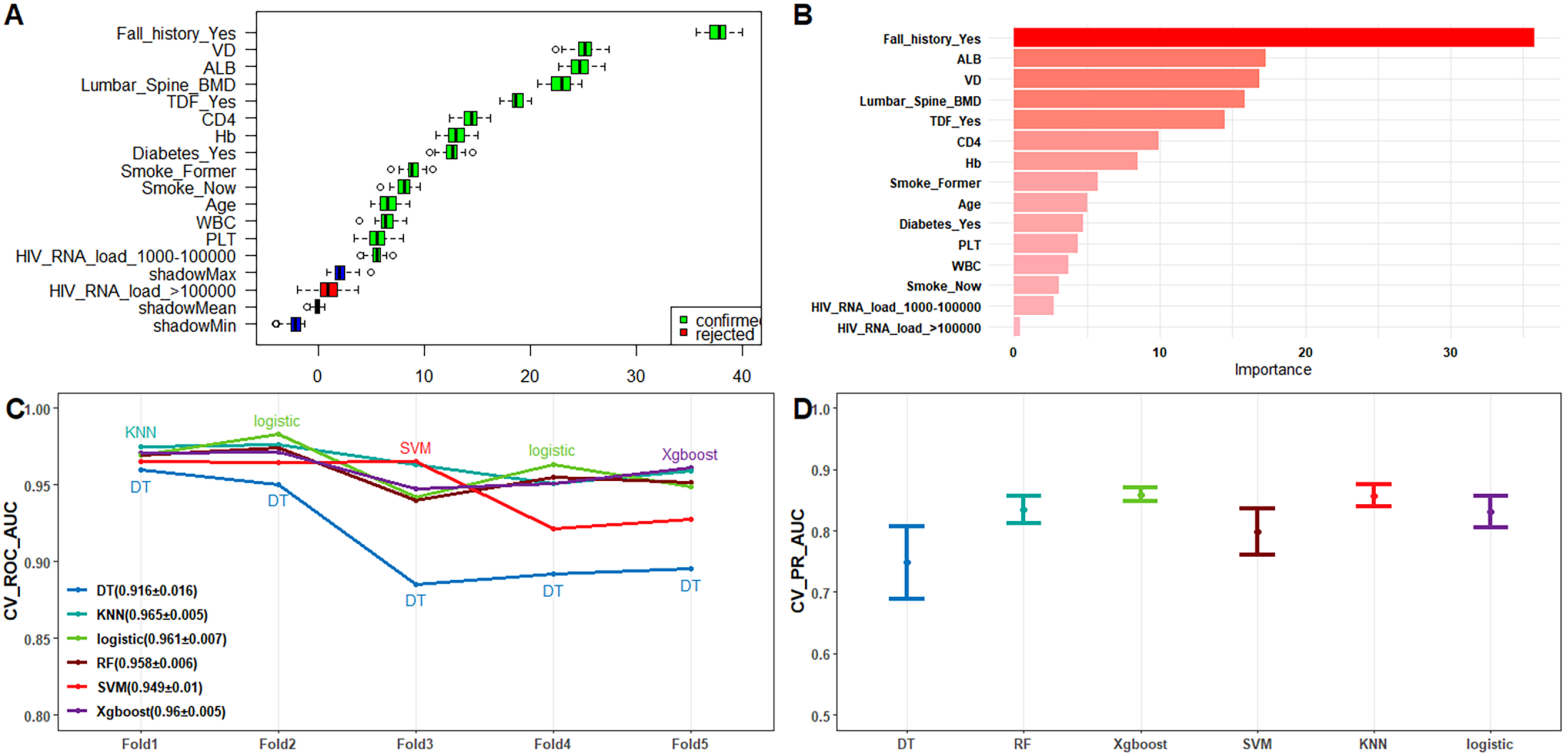
**(A)** Feature screening using Boruta’s algorithm; **(B)** Feature importance ranking plot using RFE-RF; **(C)** Line plot of ROC-AUC for each model with 10-fold cross-validation on the training dataset; **(D)** 95% confidence intervals of the PR-AUC for each model obtained by 10-fold cross-validation on the training dataset.

To further refine our model, we employed LASSO regression analysis, which selected the following variables: CD4 count, Hb, platelet count (PLT), ALB, VD, lumber spine BMD, current smoking status, diabetes, history of falls, TDF usage, and HIV RNA load (1000-100000 copies/mL) (**Supplementary Figure 1**). This step ensured that we captured the most relevant predictors while reducing potential multicollinearity. Additionally, we utilized the Boruta algorithm (**Figure 2A**) and Recursive Feature Elimination with Random Forest (RFE-RF, **Figure 2B**) to validate and cross-check our feature selection process.

This comprehensive approach, supported by existing literature, confirmed that the top 10 most important variables for our final model were: *Age, current smoking status, diabetes, history of falls, TDF usage, HIV RNA load, CD4, white blood cell count (WBC), Hb, and lumbar* sp*ine BMD*. This meticulous feature selection process ensured that our model was both efficient and accurate, providing robust predictive capabilities for identifying the risk of fragility fractures in PLWH.

### Development and evaluation of a diagnostic model in training dataset and external test dataset

**Figure 3:**
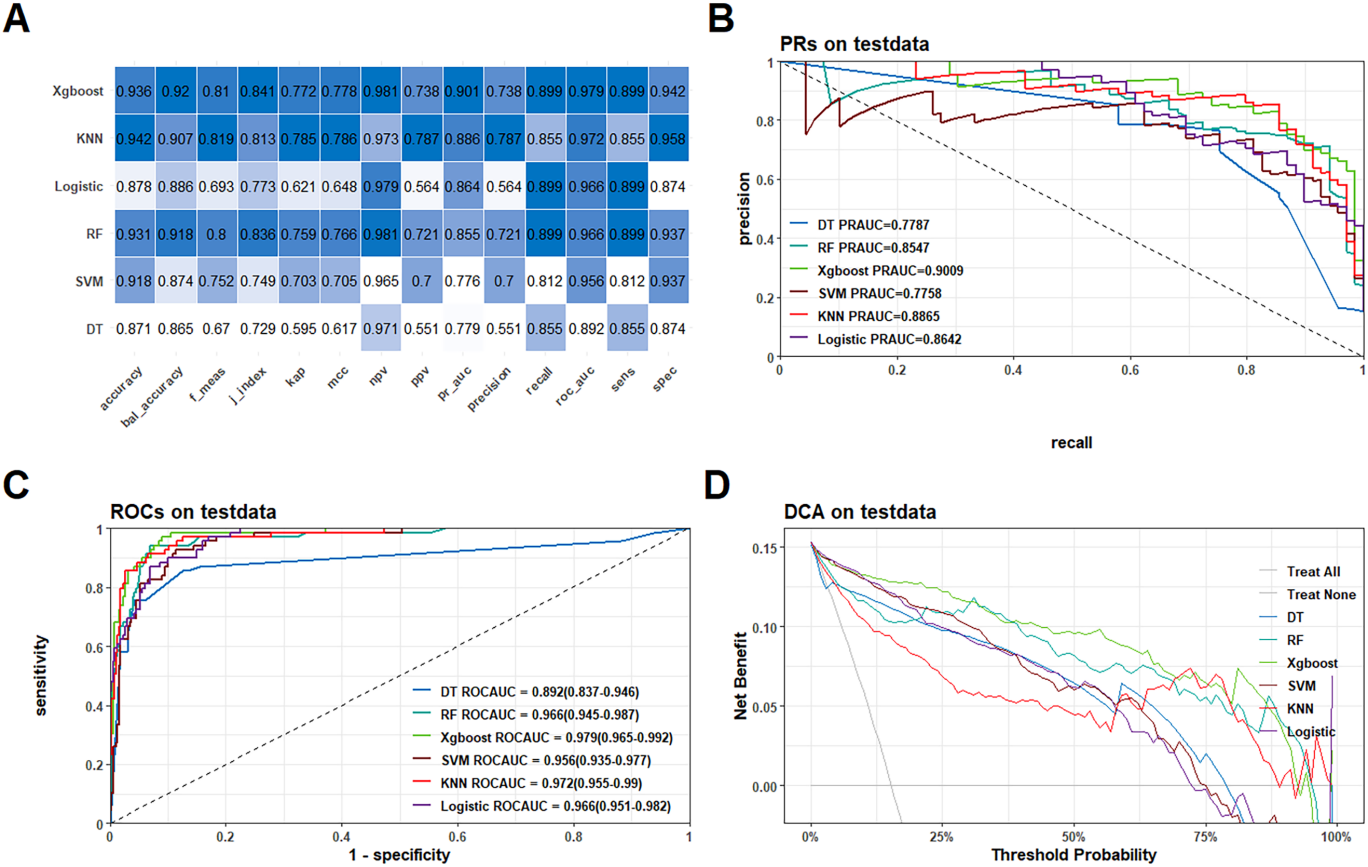
Performance comparison of the six models in the external test queue. **(A)** Heatmaps of each metric for the six models; **(B)** PR curves for the six models; **(C)** ROC curves for the six models; **(D)** DCA curves for the six models.

In the model training, a positive class represented the presence of fragility fractures, while a negative class indicated the absence of such fractures in PLWH. In the training dataset, the models demonstrated high discriminative ability as evidenced by their ROC-AUC scores: DT achieved 0.941 (95% CI: 0.918−0.964), RF 0.97 (95% CI: 0.96−0.98), XGBoost 0.984 (95% CI: 0.977−0.99), SVM 0.965 (95% CI: 0.953−0.978), KNN 0.982 (95% CI: 0.974−0.991), and Logistic Regression 0.967 (95% CI: 0.957−0.977) (**Supplementary Figure 2C**). These models also performed well in the external test dataset with the following ROC-AUC scores: DT 0.892 (95% CI: 0.837−0.946), RF 0.966 (95% CI: 0.945−0.987), XGBoost 0.979 (95% CI: 0.965−0.992), SVM 0.956 (95% CI: 0.935−0.977), KNN 0.972 (95% CI: 0.955−0.99), and LR 0.966 (95% CI: 0.951−0.982) (**Figure 3C**). The PR-AUC metrics further supported the robustness of these models. In the training dataset, the PR-AUC scores were: DT 0.8335, RF 0.8786, XGBoost 0.9275, SVM 0.8269, KNN 0.9326, and Logistic Regression 0.8411 **(Supplementary Figure 2B)**. In the external test dataset, the PR-AUC scores were: DT 0.7787, RF 0.8547, XGBoost 0.9009, SVM 0.7758, KNN 0.8865, and Logistic Regression 0.8642 (**Figure 3B**).

Among these models, the XGBoost algorithm demonstrated the best overall performance. In the training dataset, the XGBoost model achieved a ROC-AUC of 0.984 and a PR-AUC of 0.9275. In the external test dataset, it achieved a ROC-AUC of 0.979 and a PR-AUC of 0.9009, indicating strong predictive power and generalizability (**Figures 2C, D**). Further the calibration curves and Decision Curve Analysis (DCA) reinforced the reliability of the XGBoost model. The calibration curves plot indicated good agreement between predicted and observed probabilities of fragility fractures (**Supplementary Figure 3**), while the DCA showed that the XGBoost model provided a high net benefit across a range of threshold probabilities (**Supplementary Figure 2D**; **Figure 3D**).

In conclusion, the development and evaluation of these diagnostic models, particularly the XGBoost model, highlighted their potential utility in accurately predicting fragility fractures among PLWH, thereby facilitating early intervention and management in this vulnerable population.

### Optimal predictive performance of the XGBoost model for fragility fractures in PLWH

As shown in **Table 3**, our study demonstrate that the XGBoost model exhibited the highest predictive performance among the six machine learning models evaluated for predicting fragility fractures in PLWH. In the training set, the XGBoost model achieved a ROC-AUC of 0.984 (95% CI: 0.977−0.99), a PR-AUC of 0.928, an accuracy of 0.944, a sensitivity (recall) of 0.924, a specificity of 0.948, a precision of 0.760, and an F1-score of 0.834. In the external test set, it achieved a ROC-AUC of 0.979 (95% CI: 0.965−0.992), a PR-AUC of 0.901, an accuracy of 0.936, a sensitivity (recall) of 0.899, a specificity of 0.942, a precision of 0.738, and an F1-score of 0.810 (**Figure 3A**).

**Table 3:** Results of diagnostic performance metrics for each model for PLWH fragility fractures in the training set and external test dataset.

Dataset	Model	ROC_AUC	PR_AUC	Accuracy	Sensitivity	Specificity	Precision	Recall	F1-score
**Traindata**	**DT**	0.941	0.833	0.900	0.911	0.897	0.613	0.911	0.733
	**RF**	0.970	0.879	0.922	0.911	0.923	0.679	0.911	0.778
	**Xgboost**	0.984	0.928	0.944	0.924	0.948	0.760	0.924	0.834
	**SVM**	0.965	0.827	0.918	0.911	0.919	0.667	0.911	0.770
	**KNN**	0.982	0.933	0.936	0.943	0.935	0.720	0.943	0.816
	**Logistic**	0.967	0.841	0.879	0.968	0.864	0.558	0.968	0.708
**Testdata**	**DT**	0.892	0.779	0.871	0.855	0.874	0.551	0.855	0.670
	**RF**	0.966	0.855	0.931	0.899	0.937	0.721	0.899	0.800
	**Xgboost**	0.979	0.901	0.936	0.899	0.942	0.738	0.899	0.810
	**SVM**	0.956	0.776	0.918	0.812	0.937	0.700	0.812	0.752
	**KNN**	0.972	0.886	0.942	0.855	0.958	0.787	0.855	0.819
	**Logistic**	0.966	0.864	0.878	0.899	0.874	0.564	0.899	0.693

DT, Decision Tree; RF, Random Forest; Xgboost, Extreme Gradient Boosting; SVM, Support Vector Machine; KNN, K-Nearest Neighbors; Logistic, Logistic Regression.

Bold implies that the p-value is statistically significant.

The Precision-Recall (PR) curve and Decision Curve Analysis (DCA) confirmed the model’s effectiveness and clinical utility. The PR-AUC was 0.928 in the training set and 0.901 in the external test set (**Supplementary Figures 4**, **5**), indicating a good balance between precision and recall. The DCA demonstrated a positive net benefit across various threshold probabilities, supporting the model’s practical applicability in clinical settings.

In comparison with the FRAX model, which was constructed using similar variables from our dataset, the XGBoost model outperformed the FRAX-based logistic regression model in predicting fragility fractures in PLWH. The ROC-AUC of the XGBoost model was 0.984 (95% CI: 0.977−0.99) in the training set, whereas the FRAX model achieved a ROC-AUC of 0.89 (95% CI: 0.85−0.92). These results highlight the superior predictive performance of the XGBoost model for the PLWH population, which has unique risk factors not fully captured by the FRAX model, which was developed for the general population. In summary, the XGBoost model proved to be the optimal choice for predicting fragility fracture risk in PLWH, exhibiting high performance metrics and clinical relevance. The deployment of this model as a web-based calculator provides a valuable tool for healthcare providers, facilitating early identification and intervention to reduce the burden of fragility fractures in this vulnerable population.

### Model interpretation for the XGBoost model

**Figure 4:**
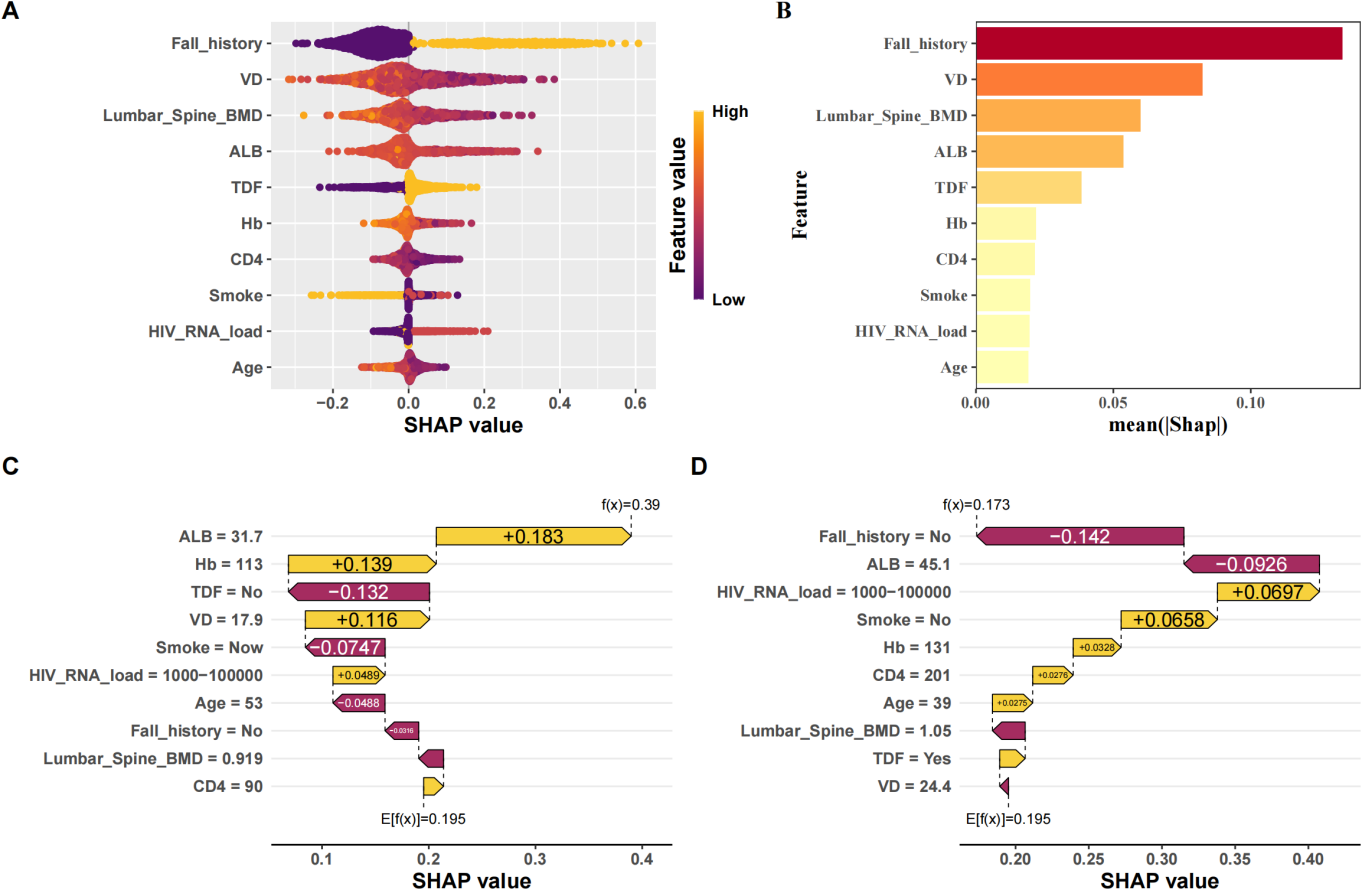
Interpretation of the best model (XGBoost) using SHAP. **(A)** SHAP beeswarm plot of features; **(B)** Ranking of feature importance by SHAP; **(C)** SHAP waterfall plot of each feature contribution for patients who did not experience fragility fractures; **(D)** SHAP waterfall plot of the contribution of each feature to patients with fragile fractures.

To ensure a comprehensive understanding of the selected variables, we employed the SHAP (SHapley Additive exPlanations) algorithm to highlight their predictive importance in the optimal XGBoost model for fragility fractures among PLWH. **Figure 4A** visually demonstrates the key features of the XGBoost model, including age, smoking status, history of falls, tenofovir disoproxil fumarate (TDF) use, HIV viral load, vitamin D levels, hemoglobin, albumin, CD4 count, and lumbar spine bone mineral density (BMD). Each dot represents a sample, with red indicating higher risk values and blue indicating lower ones. The SHAP values on the x-axis indicate the impact of each feature on the model’s output. **Figure 4B** depicts the hierarchical organization of these risk factors, underlining their significance in the model. **Figure 4C** and **Figure 4D** are SHAP force plots, providing detailed feature contributions for individual predictions. Each feature’s impact on the final prediction is illustrated with arrows, where the length and color of the arrows indicate the magnitude and direction of the feature’s effect.

**Supplementary Figure 6** provides a variable dependence plot for each feature, illustrating the relationship between each variable and the outcome variable. Specifically, we observed that all continuous variables (age, HIV viral load, vitamin D levels, hemoglobin, albumin, CD4 count, and lumbar spine BMD) were negatively correlated with the outcome, indicating they act as protective factors (**Supplementary Figure 6A**). In contrast, all categorical variables (smoking status, history of falls, and TDF use) were positively correlated with the outcome, identifying them as risk factors (**Supplementary Figure 6B**). The negative correlation of continuous variables can be attributed to their roles in maintaining bone health and immune function. Higher levels of vitamin D, hemoglobin, and albumin are associated with better bone density and strength, while higher CD4 counts and lumbar spine BMD reflect better immune function and bone health, reducing fracture risk. Conversely, the positive correlation of categorical variables such as smoking status, history of falls, and TDF use aligns with their known associations with increased fracture risk. Smoking and falls contribute to bone weakening and injury risk, while long-term TDF use has been linked to reduced bone mineral density in PLWH.

### Online web assessment tool for fragile fractures in PLWH

**Figure 5:**
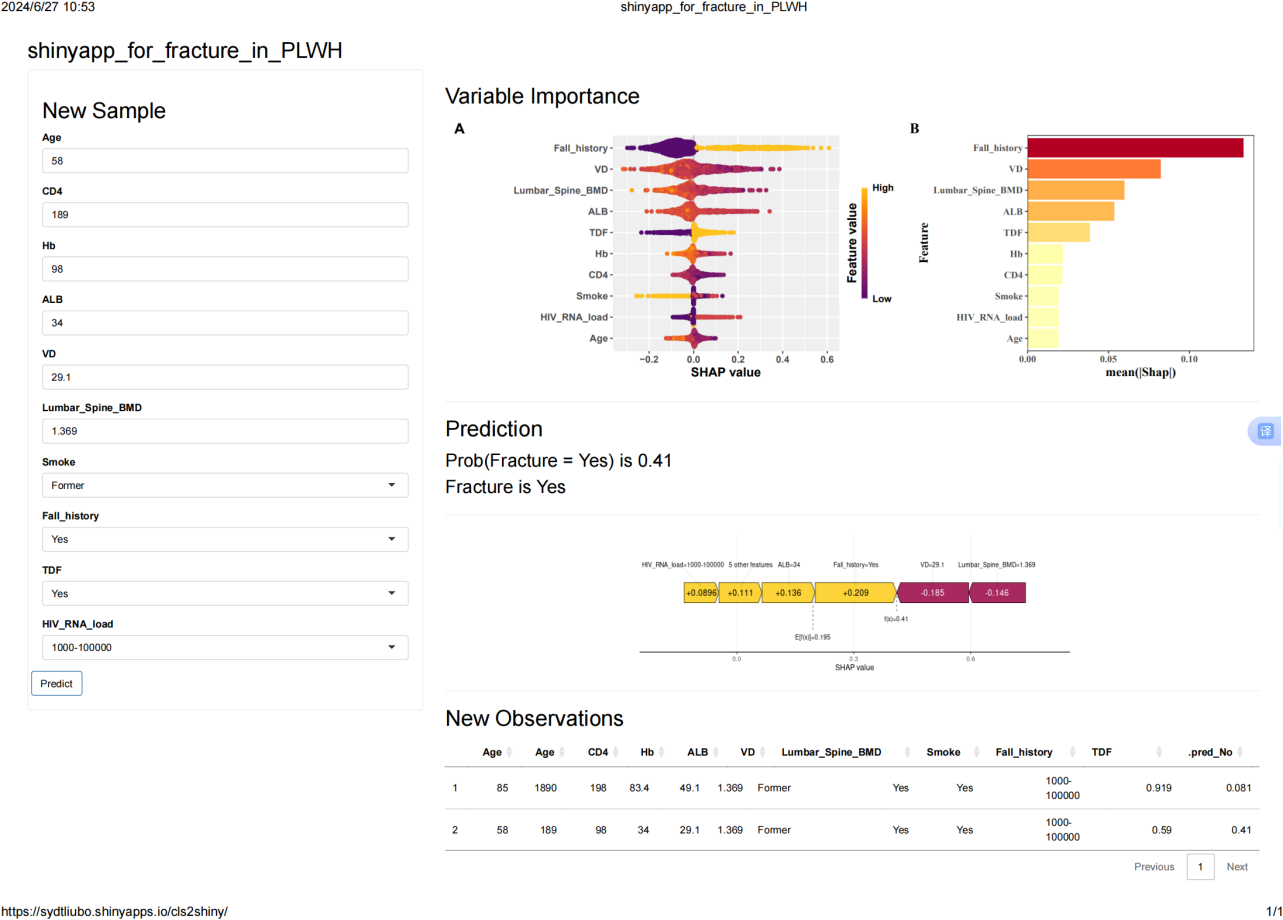
Interface of the online web application using the best XGBoost model.

The integration of the XGBoost model into a publicly accessible web-based calculator (https://sydtliubo.shinyapps.io/cls2shiny/) allows clinicians and patients to assess the risk of fragility fractures in real-time (**Figure 5**). This tool is designed to be user-friendly, providing an easy-to-understand risk assessment and detailed interpretation of each prediction, thus facilitating informed clinical decisions and potentially reducing the burden of fragility fractures among PLWH.

## Discussion

In this study, we aimed to develop and validate a web-based risk assessment calculator using machine learning algorithms to predict the risk of fragility fractures in PLWHs. The XGBoost machine learning model demonstrated excellent predictive performance in assessing fragility fracture risk. It achieved an area under the receiver operating characteristic curve (ROC-AUC) of 0.984 (95% CI: 0.977−0.99) in the training set and 0.979 (95% CI: 0.965−0.992) in the external test set. These results indicate the model’s ability to accurately predict fracture risk in PLWH. Through feature selection, we identified several key risk factors associated with fragility fractures in PLWH. These factors include age, smoking, fall history, tenofovir disoproxil fumarate (TDF) use, HIV viral load, vitamin D levels, hemoglobin levels, albumin levels, CD4 count, and lumbar spine bone mineral density (BMD). By considering these factors, the web-based calculator can provide a comprehensive assessment of fracture risk.

To the best of our knowledge, this study is the first to develop a web-based calculator specifically tailored for predicting fragility fracture risk in PLWH. By leveraging machine learning algorithms, our model outperformed previous studies in fracture risk prediction (Vizcarra et al., 2023). Existing literature on fracture risk prediction mainly focuses on the general population or specific subgroups, often excluding PLWH. The unique challenges faced by PLWH, including increased fracture risk and associated morbidity and mortality, necessitate a tailored approach. Our study addresses this gap by providing a specialized tool that considers both traditional and HIV-specific risk factors. Furthermore, our study contributes to the field by incorporating interpretable predictions through SHAP values. These values allow clinicians to understand the influence of each risk factor on the predicted fracture risk, enabling personalized risk stratification and management (Uragami et al., 2023). The web-based calculator developed in this study fills an important void in clinical practice by providing a user-friendly tool for fracture risk assessment in PLWH. Its integration into existing clinical workflows and guidelines has the potential to reduce the burden of fragility fractures in this population and improve patient outcomes.

Several fracture risk prediction tools, such as FRAX (Fracture Risk Assessment Tool) and QFracture, have been developed and widely used in the general population (Kanis et al., 2008; van den Bergh et al., 2010; Kanis et al., 2016). However, these tools have limited applicability to PLWH due to their failure to account for the unique risk factors associated with HIV infection and antiretroviral therapy (ART). These HIV-specific factors, including viral load, CD4 count, and the direct and indirect effects of ART on bone metabolism, play a crucial role in the heightened fracture risk observed in this population (Yin and Falutz, 2016; McGee and Cotter, 2024). To address this gap, a web-based risk assessment calculator has been developed utilizing machine learning algorithms, specifically the powerful XGBoost model. This calculator is tailored to the specific risk factors of PLWH by incorporating both HIV-specific factors (viral load, CD4 count) and traditional fracture risk factors (age, gender, smoking, vitamin D levels, bone mineral density, etc.). By considering this comprehensive set of relevant variables and their complex interactions, the calculator can provide more accurate and personalized fracture risk assessments for PLWH.

The heightened vulnerability to fragility fractures among those living with HIV is driven by an intricate interplay of various interconnected elements. Aging itself predisposes individuals to bone loss and fractures, a condition exacerbated by the direct and indirect impacts of HIV infection and its treatments (Mallon, 2014). Smoking exerts detrimental effects on bone metabolism, compounding the risk when combined with HIV-related factors (Boyer et al., 2023). A history of falls, which can precipitate fragility fractures, is more common due to HIV-associated conditions like muscle wasting, neuropathy, and medication side effects (Womack et al., 2021). Certain antiretroviral drugs, notably tenofovir disoproxil fumarate (TDF), have been linked to decreased bone mineral density (BMD) and heightened fracture susceptibility, potentially through nephrotoxic mechanisms that impair bone metabolism (Delpino and Quarleri, 2020). The HIV virus itself can contribute to bone loss through the direct effects of viral proteins on bone cells, as well as indirectly via chronic inflammation and immune dysregulation associated with higher viral loads and lower CD4 counts (Ofotokun et al., 2012; Lewy et al., 2019). Nutritional deficiencies, such as vitamin D deficiency and low hemoglobin and albumin levels, which reflect overall health status, further compromise bone health (Hileman et al., 2016). Ultimately, low BMD, particularly in the lumbar spine, serves as a direct measure of bone strength and a powerful predictor of fracture risk in this population (Chang et al., 2021). This multifaceted interplay of age-related, HIV-specific, and traditional osteoporosis risk factors converges to amplify the vulnerability of PLWH to fragility fractures, necessitating a comprehensive and personalized approach to risk assessment and management (Negredo et al., 2016).

The XGBoost model, a powerful ensemble learning algorithm, can effectively capture complex nonlinear relationships and interactions between the diverse risk factors associated with fragility fractures in PLWH (Uragami et al., 2023; Wu and Park, 2023). By incorporating HIV-specific variables (viral load, CD4 count, ART regimen) alongside demographic, clinical, and lifestyle factors, an optimized XGBoost model can provide highly accurate and personalized fracture risk assessments. A key advantage of XGBoost is its interpretability, facilitated by techniques like SHAP. SHAP offers a unified approach to explaining the output of machine learning models by quantifying the contributions of each feature to the model’s predictions (Belle and Papantonis, 2021). SHAP can unravel the intricate interplay between HIV-related factors and traditional fracture risk factors, revealing their relative importance and potential interactions. For example, SHAP could help identify subgroups of PLWH with specific combinations of risk factors (e.g., lower CD4 count, prolonged ART exposure, vitamin D deficiency) that render them particularly vulnerable to fragility fractures (Premaor and Compston, 2020). By visualizing SHAP values, clinicians can gain insights into the most influential risk factors for each individual patient, enabling tailored interventions and preventive strategies. Moreover, SHAP can elucidate the complex, non-linear relationships between predictors and fracture risk, which may be difficult to discern using traditional statistical methods. This improved interpretability can enhance clinical decision-making, foster trust in the machine learning model, and ultimately contribute to better fracture risk management in the PLWH. While the XGBoost model offers superior predictive performance, the integration of SHAP-based interpretability is crucial for translating these predictions into actionable clinical insights, ensuring the responsible and ethical deployment of machine learning in healthcare settings.

However, it is important to acknowledge the limitations of our study. As a retrospective study, there is a potential risk of selection bias and unmeasured confounding factors. Prospective validation in a larger, multi-center cohort would further strengthen the generalizability of our findings. Furthermore, while our dataset included a comprehensive set of risk factors, it is possible that additional factors, such as genetic markers or biomarkers, could further improve the predictive performance of the model. Future studies incorporating these additional variables may enhance the accuracy of fracture risk assessment. Finally, it is essential to note that our study focused specifically on PLWH. While the calculator is tailored for this population, its applicability to other high-risk groups or the general population may be limited and requires further investigation. Additionally, our study did not incorporate time-dependent variables, which are critical in models like FRAX. While the FRAX model uses a Cox proportional hazards model with time variables, our machine learning-based XGBoost model is a diagnostic model without time as a factor. The potential use of survival models that incorporate time variables could be an interesting direction for future research.

## Conclusion

Our study successfully developed and validated a novel web-based risk assessment tool for predicting fragility fractures in PLWH using machine learning algorithms. The XGBoost model demonstrated superior predictive performance, achieving high discrimination and calibration in both the training and external test sets. The model incorporated clinically relevant features, including age, smoking status, fall history, antiretroviral therapy, HIV viral load, vitamin D levels, hemoglobin, albumin, CD4 count, and lumbar spine BMD. The user-friendly web calculator, powered by the XGBoost algorithm, provides a valuable resource for clinicians and patients to assess fracture risk and guide preventive measures. The interpretability of the model’s predictions through SHAP values further enhances its clinical utility by explaining individual risk profiles. This web-based tool has the potential to improve fracture risk stratification and management in the PLWH, ultimately reducing the burden of fragility fractures and associated complications. In conclusion, our study presents a significant advancement in fracture risk prediction for PLWH. The contribution of our study lies in addressing a significant gap in clinical practice by providing a specialized tool tailored for fracture risk assessment in PLWH. By considering both traditional and HIV-specific risk factors, our web-based calculator offers a comprehensive approach to identifying high-risk patients and informing fracture prevention and management strategies.

## Data Availability

The original contributions presented in the study are included in the article/**Supplementary Material.** Further inquiries can be directed to the corresponding authors.

## Supplementary material

Supplementary Figure 1 Feature selection using the Least Absolute Shrinkage and Selection Operator (LASSO). **(A)** By verifying the optimal parameter (lambda) in the LASSO model, the partial likelihood deviance (binomial deviance) curve was plotted versus log(lambda) and dotted vertical lines were drawn based on 1 standard error criteria.11 variables with nonzero coefficients were selected by optimal lambda; **(B)** coefficient profile plot was produced against the log(lambda) sequence.

Supplementary Figure 2 Performance comparison of the six models on the training dataset. **(A)** Heatmaps of each metric for the six models; **(B)** PR curves for the six models; **(C)** ROC curves for the six models; **(D)** DCA curves for the six models.

Supplementary Figure 3 Calibration plots of the six models in the training dataset **(A)** and external test dataset **(B)**.

Supplementary Figure 4 Evaluation metrics of the best model (XGBoost) in the training set. **(A)** ROC curve of XGBoost model; **(B)** Confusion matrix of XGBoost model; **(C)** PR curve of XGBoost model; **(D)** KS curve of XGBoost model.

Supplementary Figure 5 Evaluation of the best model (XGBoost) on the external test set. **(A)** ROC curve of XGBoost model; **(B)** Confusion matrix of XGBoost model; **(C)** PR curve of XGBoost model; **(D)** KS curve of XGBoost model.

Supplementary Figure 6 SHAP values for each variable. **(A)** SHAP values for continuous variables (vitamin D, lumbar spine bone density, albumin, hemoglobin, CD4 and age); **(B)** SHAP values for categorical variables (history of falls, TDF use, smoking and HIV viral load). A positive SHAP value means likely to have a fracture; a negative value means unlikely to have a fracture. SHAP, Shapley additive explanations.
