# Supplementary figures and images for: An explainable web application based on machine learning for predicting fragility fracture in people living with HIV: data from Beijing Ditan Hospital, China
Source: Front Cell Infect Microbiol. 2025 Mar 14;15:1461740. doi: 10.3389/fcimb.2025.1461740 (PMC11949899; doi:10.3389/fcimb.2025.1461740)

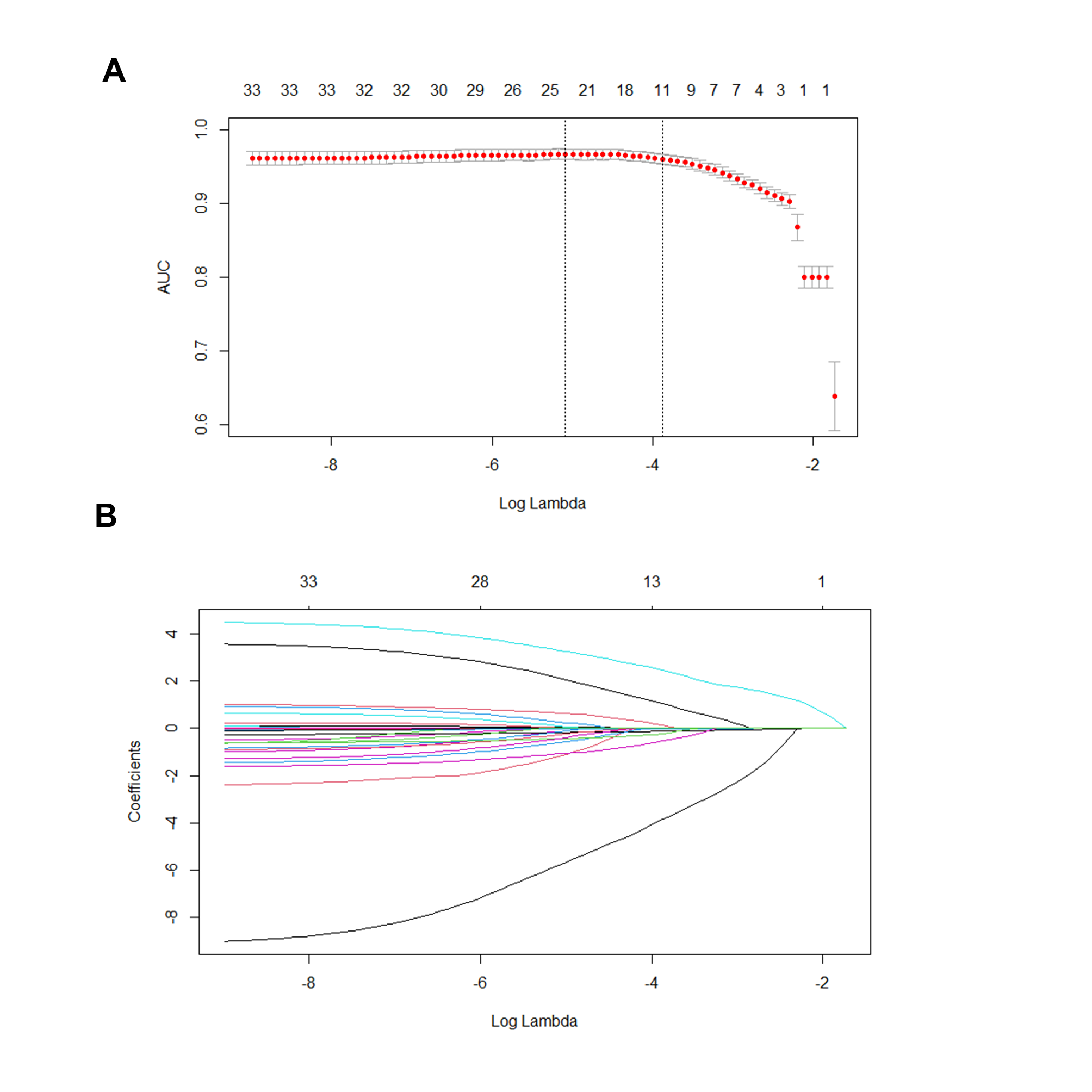

Supplement: Supplementary Figure 1 — Feature selection using the Least Absolute Shrinkage and Selection Operator (LASSO). (A) By verifying the optimal parameter (lambda) in the LASSO model, the partial likelihood deviance (binomial deviance) curve was plotted versus log(lambda) and dotted vertical lines were drawn based on 1 standard error criteria.11 variables with nonzero coefficients were selected by optimal lambda; (B) coefficient profile plot was produced against the log(lambda) sequence. [file Image1.tif]

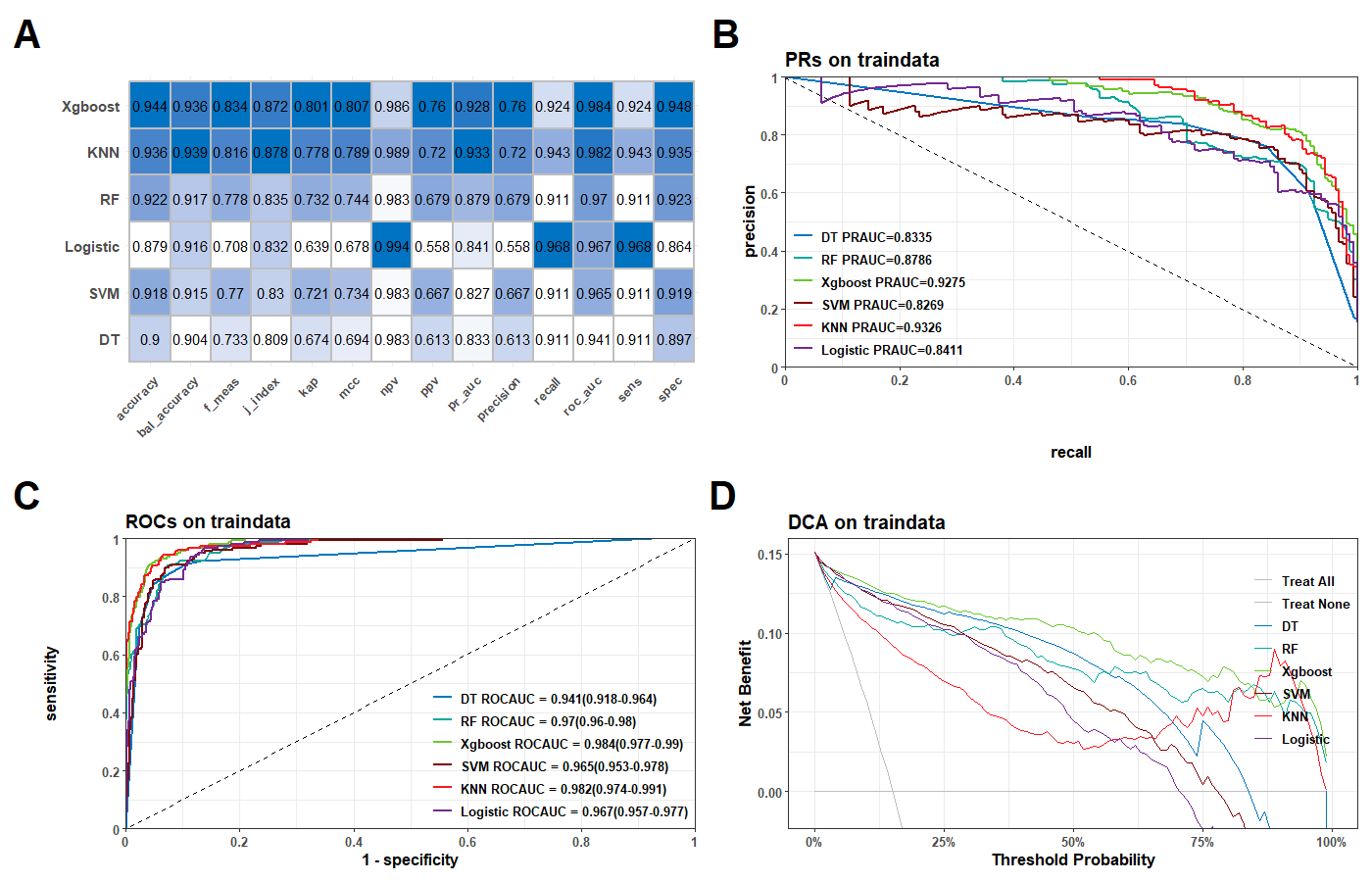

Supplement: Supplementary Figure 2 — Performance comparison of the six models on the training dataset. (A) Heatmaps of each metric for the six models; (B) PR curves for the six models; (C) ROC curves for the six models; (D) DCA curves for the six models. [file Image2.tiff]

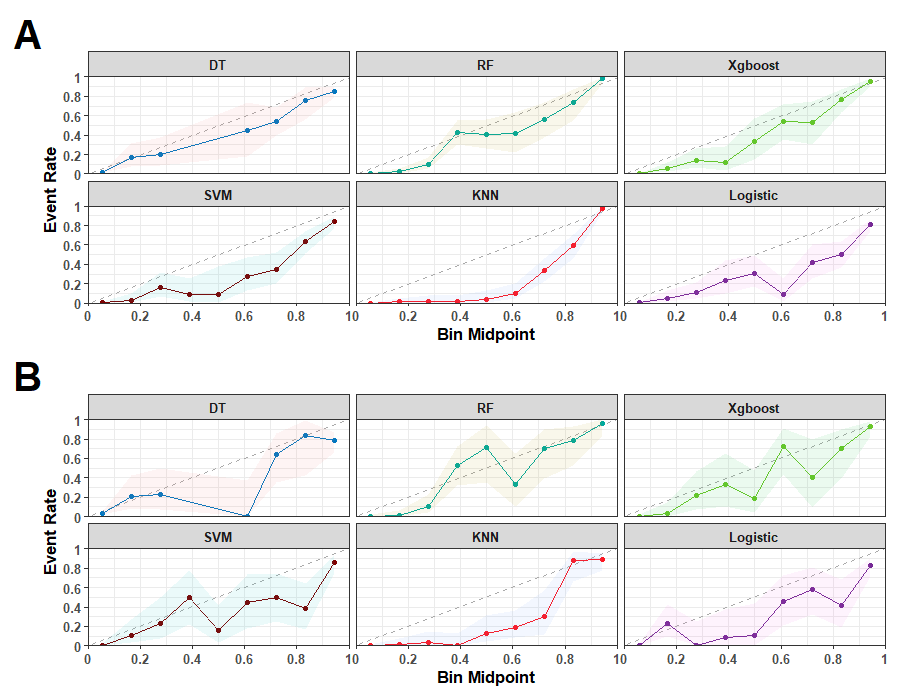

Supplement: Supplementary Figure 3 — Calibration plots of the six models in the training dataset (A) and external test dataset (B). [file Image3.tiff]

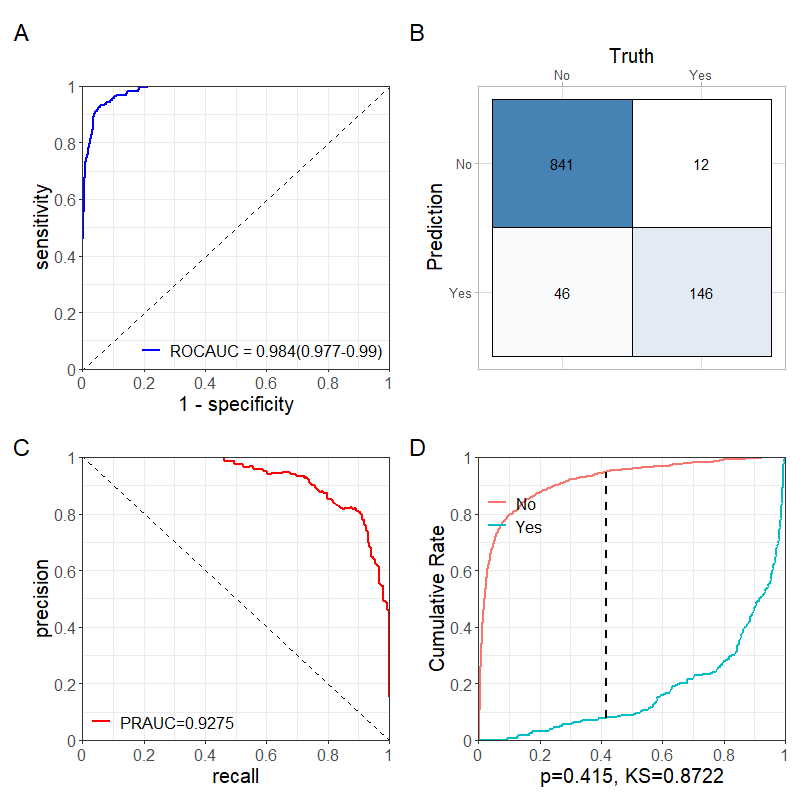

Supplement: Supplementary Figure 4 — Evaluation metrics of the best model (XGBoost) in the training set. (A) ROC curve of XGBoost model; (B) Confusion matrix of XGBoost model; (C) PR curve of XGBoost model; (D) KS curve of XGBoost model. [file Image4.tiff]

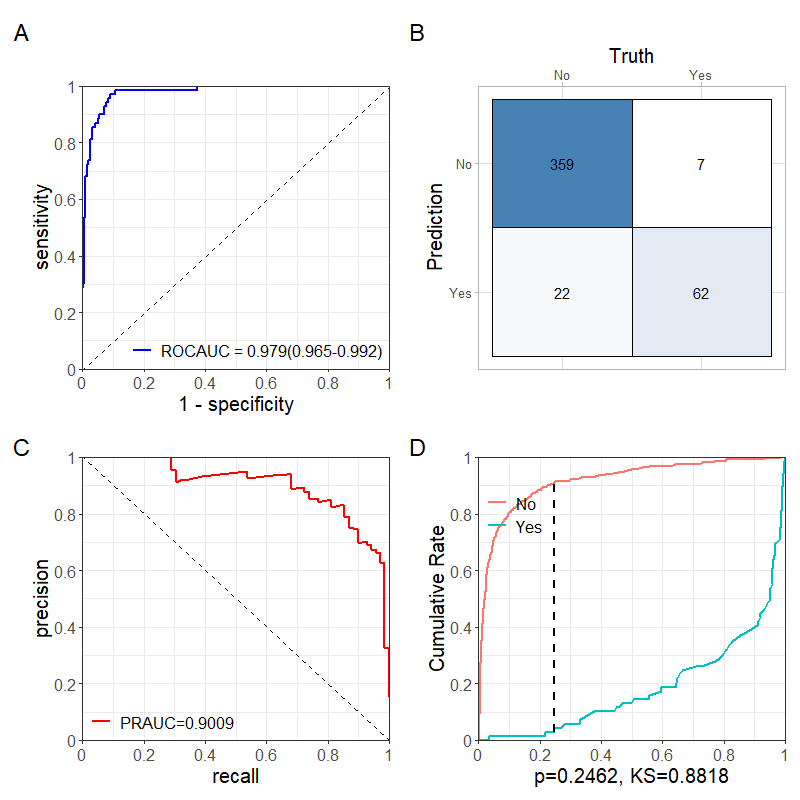

Supplement: Supplementary Figure 5 — Evaluation of the best model (XGBoost) on the external test set. (A) ROC curve of XGBoost model; (B) Confusion matrix of XGBoost model; (C) PR curve of XGBoost model; (D) KS curve of XGBoost model. [file Image5.tiff]

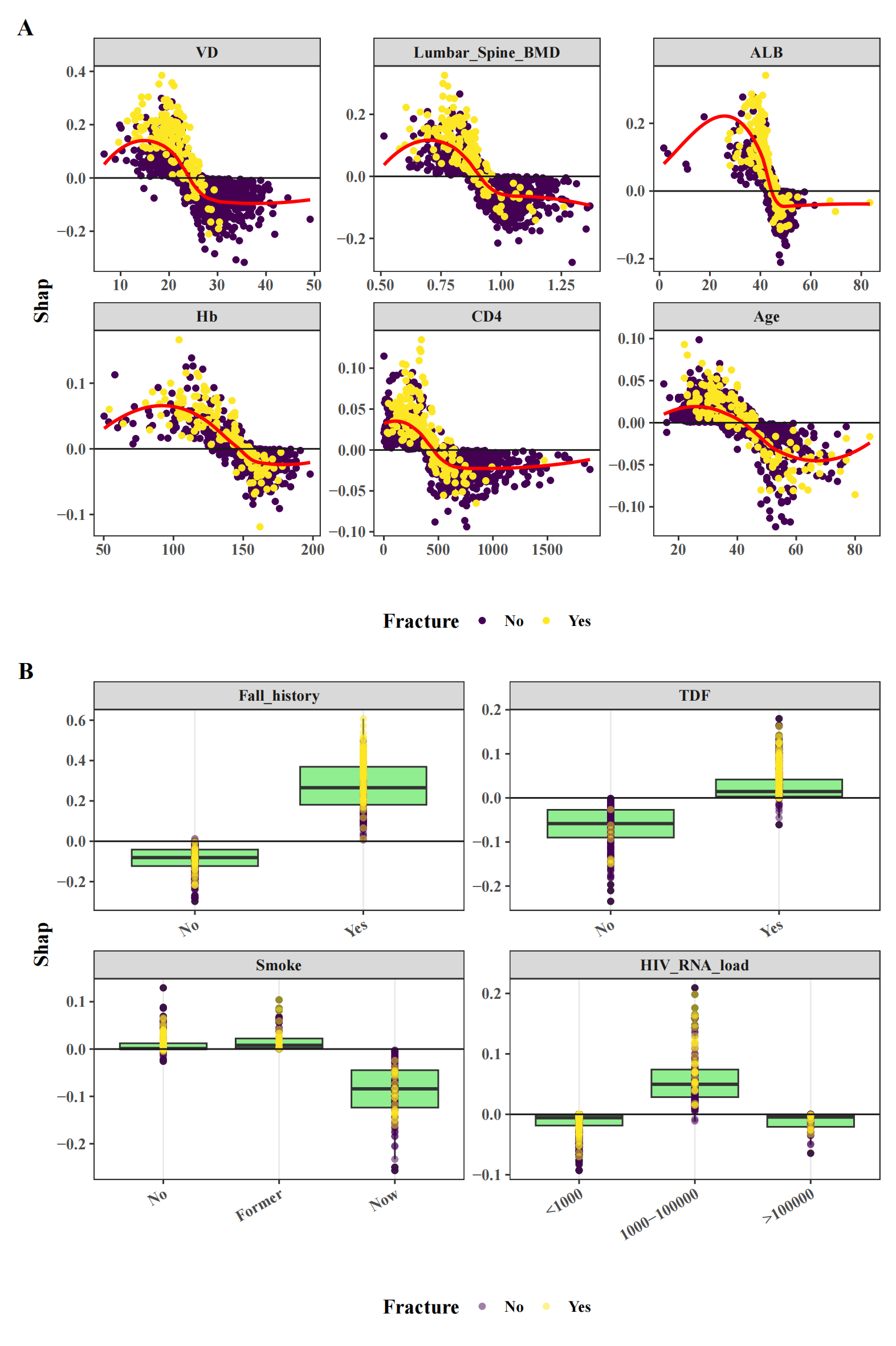

Supplement: Supplementary Figure 6 — SHAP values for each variable. (A) SHAP values for continuous variables (vitamin D, lumbar spine bone density, albumin, hemoglobin, CD4 and age); (B) SHAP values for categorical variables (history of falls, TDF use, smoking and HIV viral load). A positive SHAP value means likely to have a fracture; a negative value means unlikely to have a fracture. SHAP, Shapley additive explanations. [file Image6.tif]
