# Supplementary material for: An explainable web application based on machine learning for predicting fragility fracture in people living with HIV: data from Beijing Ditan Hospital, China
Source: Front Cell Infect Microbiol. 2025 Mar 14;15:1461740. doi: 10.3389/fcimb.2025.1461740 (PMC11949899; doi:10.3389/fcimb.2025.1461740)
Supplement: Supplementary file 7 [file Table1.docx]

**Supplementary Table 1 Baseline characteristics of the training dataset and external test dataset for HIV-positive patients.**

| **Variables** | **All** | **Train dataset** | **Test dataset** | **P-value** |
| --- | --- | --- | --- | --- |
|  | ***N=1495*** | ***N=1045*** | ***N=450*** |  |
| Fracture (n, %) | 227 (15.2%) | 158 (15.1%) | 69 (15.3%) | 0.978 |
| Gender: Male (n, %) | 1372 (91.8%) | 960 (91.9%) | 412 (91.6%) | 0.922 |
| Age (Years) | 38.9 (11.5) | 39.2 (11.6) | 38.4 (11.2) | 0.192 |
| Menopause (n, %) | 65 (52.8%) | 48 (56.5%) | 17 (44.7%) | 0.313 |
| BMI (kg/m^2^) | 23.1 (3.46) | 23.1 (3.40) | 23.2 (3.60) | 0.771 |
| Smoke (n, %) |  |  |  | 0.046 |
| Former | 111 (7.42%) | 89 (8.52%) | 22 (4.89%) |  |
| Now | 187 (12.5%) | 127 (12.2%) | 60 (13.3%) |  |
| Drinking (n, %) | 300 (20.1%) | 213 (20.4%) | 87 (19.3%) | 0.693 |
| Hypertension (n, %) | 174 (11.6%) | 128 (12.2%) | 46 (10.2%) | 0.302 |
| Diabetes (n, %) | 266 (17.8%) | 176 (16.8%) | 90 (20.0%) | 0.164 |
| HBV and/or HCV (n, %) | 195 (13.0%) | 130 (12.4%) | 65 (14.4%) | 0.331 |
| Fall_history (n, %) | 387 (25.9%) | 271 (25.9%) | 116 (25.8%) | 1.000 |
| Corticosteroids_used (n, %) | 180 (12.0%) | 135 (12.9%) | 45 (10.0%) | 0.133 |
| Duration_infection (month) | 57.7 (47.1) | 58.8 (47.7) | 55.1 (45.4) | 0.159 |
| TDF (n, %) | 1037 (69.4%) | 736 (70.4%) | 301 (66.9%) | 0.193 |
| HIV_RNA_load (n, %) |  |  |  | 0.495 |
| 1000-100000 | 245 (16.4%) | 168 (16.1%) | 77 (17.1%) |  |
| >100000 | 117 (7.83%) | 77 (7.37%) | 40 (8.89%) |  |
| CD4 (cells/ul) | 544 (309) | 543 (305) | 547 (317) | 0.815 |
| CD8 (cells/ul) | 924 (509) | 922 (506) | 929 (516) | 0.802 |
| CD4_CD8_Ratio | 0.68 (0.46) | 0.68 (0.42) | 0.69 (0.56) | 0.571 |
| WBC (10^9^/L) | 6.20 (2.05) | 6.21 (2.12) | 6.17 (1.90) | 0.719 |
| Hb (g/L) | 148 (20.5) | 149 (20.4) | 147 (20.5) | 0.251 |
| PLT (10^9^/L) | 230 (65.5) | 232 (66.6) | 226 (62.8) | 0.094 |
| ALB (g/L) | 45.8 (5.42) | 45.8 (5.53) | 45.8 (5.15) | 0.868 |
| Ca (mmol/l) | 2.31 (0.12) | 2.31 (0.12) | 2.31 (0.12) | 0.824 |
| P (mmol/l) | 1.02 (0.29) | 1.03 (0.33) | 1.00 (0.18) | 0.023 |
| VD (ng/mL) | 25.3 (6.05) | 25.4 (6.04) | 25.1 (6.07) | 0.300 |
| TC (mmol/l) | 4.40 (0.94) | 4.39 (0.93) | 4.41 (0.98) | 0.837 |
| TG (mmol/l) | 1.64 (1.00) | 1.64 (1.02) | 1.63 (0.96) | 0.865 |
| LDL_C (mmol/l) | 2.70 (1.07) | 2.70 (1.18) | 2.67 (0.76) | 0.527 |
| HDL_C (mmol/l) | 1.12 (0.32) | 1.13 (0.32) | 1.11 (0.30) | 0.130 |
| UA (umol/l) | 379 (100) | 381 (100) | 373 (101) | 0.164 |
| eGFR (ml/min/1.73m^2^) | 108 (17.3) | 108 (17.4) | 108 (17.2) | 0.684 |
| LS_BMD (g/cm^3^) | 0.945 (0.128) | 0.943 (0.129) | 0.949 (0.127) | 0.474 |
| LFN _BMD (g/cm^3^) | 0.766 (0.123) | 0.764 (0.122) | 0.769 (0.125) | 0.530 |
| Hip_BMD (g/cm^3^) | 0.889 (0.132) | 0.885 (0.124) | 0.896 (0.149) | 0.168 |

BMI - Body Mass Index; HBV - Hepatitis B Virus; HCV - Hepatitis C Virus；TDF - Tenofovir Disoproxil Fumarate；HIV_RNA_load - HIV RNA Load；CD4 - CD4 T Cells；CD8 - CD8 T Cells；CD4_CD8_Ratio - CD4/CD8 Ratio；WBC - White Blood Cell Count；Hb - Hemoglobin；PLT - Platelet Count；ALB - Albumin；Ca - Calcium；P - Phosphorus；VD - Vitamin D；TC - Total Cholesterol；TG - Triglycerides；LDL_C - Low-Density Lipoprotein Cholesterol；HDL_C - High-Density Lipoprotein Cholesterol；UA - Uric Acid；eGFR - Estimated Glomerular Filtration Rate；LS_BMD - Lumbar Spine Bone Mineral Density；LFN_BMD - Left Femoral Neck Bone Mineral Density；Hip_BMD - Hip Bone Mineral Density
